# Vesicles for Signal Amplification in a Biosensor for the Detection of Low Antigen Concentrations

**DOI:** 10.3390/s8127894

**Published:** 2008-12-05

**Authors:** Dorothee Grieshaber, Victoria de Lange, Thomas Hirt, Zhihua Lu, Janos Vörös

**Affiliations:** 1 ETH Zurich, Laboratory of Biosensors and Bioelectronics, Institute for Biomedical Engineering, Gloriastrasse 35, CH-8092 Zurich, Switzerland; 2 BioCure Inc., 2975 Gateway Dr. Suite 100, Norcross, 30071, GA USA E-mails: grieshaber@biomed.ee.ethz.ch; vdelange@student.ethz.ch; thirt@biocure.com; alu@biocure.com; janos.voros@biomed.ee.ethz.ch

**Keywords:** High sensitivity, antigen detection, biosensor, lipid vesicles, quartz crystal mi-crobalance

## Abstract

The sensitivity of biosensors is often not sufficient to detect diagnostically relevant biomarker concentrations. In this paper we have utilized a Quartz Crystal Microbalance with Dissipation monitoring (QCM-D) to detect dissipative losses induced by the attachment of intact vesicles. We modified a sandwich assay by coupling the secondary antibodies to vesi-cles. This resulted in an increase of detection sensitivity, achieving a diagnostically relevant detection limit of 5 ng/ml or 30 pM antigens. In addition, we could combine the individual assay steps to decrease the total time to result in about 30 minutes.

## 1. Introduction

Because of their ability to detect reagents in very low concentrations, biosensors have a big potential in early diagnostics of cancer and other diseases [1]. Currently, an increasing number of signalling pro-teins are being identified, e.g. for cancer [2] or for the Alzheimer's disease [3]. Normally, biosensors are based on the sandwich principle, where primary antibodies are adsorbed onto the surface and specifically capture antigens. In fluorescent assays the antigen concentration is then determined by a fluorescently labelled secondary antibody, specifically binding to the antigen [4]. In enzyme-linked immunosorbent assays (ELISAs) the secondary antibody is equipped with an enzyme which performs an enzymatic reaction that in most sensors leads to a colour change or, in fewer cases, produces electrons [5, 6]. Fur-thermore, these immunoassays can be miniaturized and parallelized to achieve high-throughput devices [4, 7–10].

Methods which require no fluorescent or enzymatic labels are the quartz crystal microbalance with dissipation monitoring (QCM-D) or surface plasmon resonance (SPR). The latter has been used with e.g. colloidal labels [11, 12]. With the QCM-D technique surface adsorption can be measured *in situ*. An example of an assay using the QCM-D technique in biosensing was presented by Larsson *et al.* who built a sensor where cholera toxin, bound to vesicles via its natural membrane receptor, is detected by his-tags on a lipid bilayer [13]. Another approach for achieving the required sensitivity includes increasing the surface area by adding nanotubes, as recently reported by Okuno *et al.* However, an incubation time of 12 h with several steps afterwards is too time consuming for point-of-care diagnostics [14].

Even though there is a high number of approaches, depending on the application, these sensors do not always fulfil the high sensitivity requirements [9]. To go one step further and thereby reach the required sensitivity, as well as a reduced assay time, it is essential to increase the signal, reduce the background and increase the sensitivity of the detection method itself [15]. Therefore, in our approach we have tried to lower the detection limit to a sensitivity that is sufficient to assess e.g. cancer antigens such as prostate specific antigen (PSA) where the diagnostically relevant concentration is in the range of ng/ml [16]. We have combined the moderate sensitivity of the QCM-D to dissipative losses together with the specific detection strategy of a sandwich assay. In this paper we show how we achieved the above mentioned goals by using a sandwich assay with vesicles for the signal amplification. The signal of the secondary antibodies was increased by coupling them to lipid vesicles. The larger mass and especially the increased viscoelasticity of the vesicles compared to a single antibody was *in situ* monitored by QCM-D. With our model system we were able to reach a detection limit of 5 ng/ml or 30 pM.

## 2. Results

**Figure 1.**
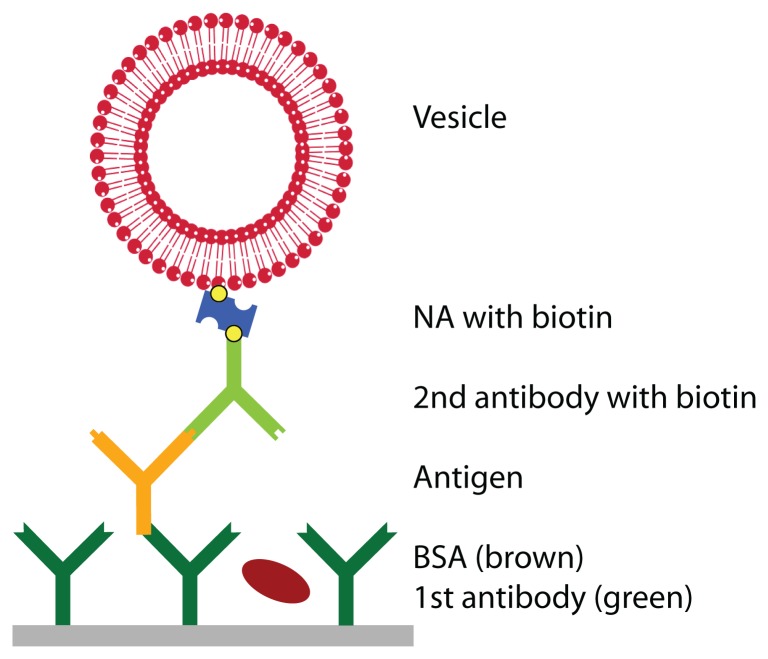
Scheme of our biosensor. The primary antibody is adsorbed to the substrate. BSA is added to prevent unspecific adsorption before the antigen is captured. Subsequently, the secondary antibody, coupled to the vesicle via biotin/neutravidin, is added.

**Figure 2.**
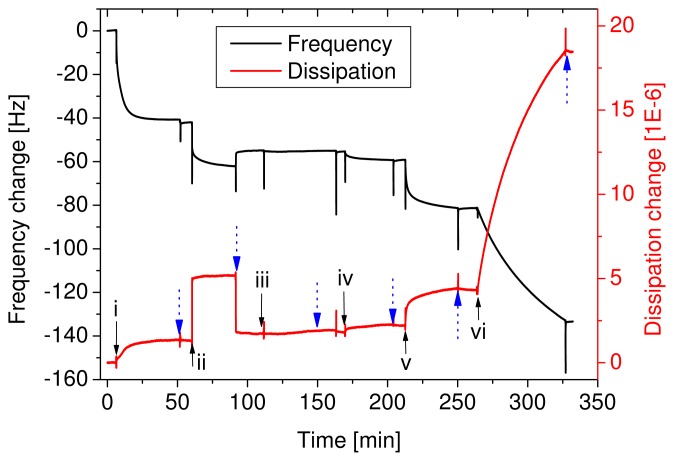
QCM-D curve showing frequency and dissipation changes during the adsorp-tion steps. i) Primary antibody, ii) BSA (part of it is removed upon rinsing), iii) antigen (400 ng/ml), iv) secondary antibody, v) neutravidin, vi) vesicles. The spikes (marked with dotted arrows) are an artefact upon injection/rinsing.

Prior to the detection of the antigen, the surface was functionalized with a primary antibody and blocked with BSA to prevent unspecific adsorption. Then, the antigen was injected at a given concentra-tion. To enhance the weak signal of the antigen, a secondary antibody, specifically binding to the antigen and functionalized with biotin, was bound, followed by the linker neutravidin and vesicles, functional-ized with biotin (see Figure 1). QCM-D curves of this adsorption sequence are shown in Figure 2. The example represents a sensor with an antigen concentration of 400 ng/ml. The adsorption of the primary antibody gave a signal in both the frequency and the dissipation change. Some BSA adsorbed as well, but upon rinsing the loosely bound molecules were rinsed off. The adsorption of the antigen is not visible in the curve because the few molecules did not yield a high enough signal. The secondary antibodies and the neutravidin resulted in a signal, but considering that 400 ng/ml antigen was far above the detec-tion limit it was quite small. Finally, the adsorption of the vesicles resulted in a big signal; a frequency change of 51 Hz and a dissipation change of 1.4E-5. Even at low antigen concentrations, which were not directly detectable with the QCM-D, the vesicles multiplied the signal and allowed for the indirect, quantitative measurement of the antigen concentration. The spikes, appearing upon injection or buffer rinse (marked with dotted arrows), are an artefact from the temporarily enhanced pressure in the flowcell and are completely reversible.

**Figure 3.**
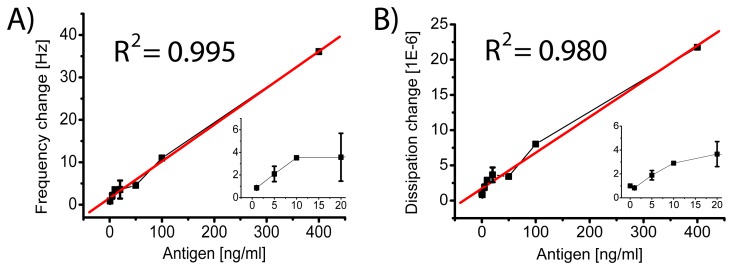
Vesicle adsorption for low antigen concentrations. A: Frequency change upon adsorption of the vesicles as a function of the antigen concentration. B: Dissipation change upon adsorption of the vesicles as a function of the antigen concentration. The insets show the detection limit at low concentrations.

For different antigen concentrations the changes in frequency and dissipation upon adsorption of the vesicles are depicted in Figure 3. For the saturation concentration, meaning the maximal number of antigens that can sterically fit on the surface, the antigens themselves still yielded a small signal in the QCM-D. However, compared to the signal from the vesicles it was not too pronounced, i.e. it was around 10-20 times smaller, depending on the different concentrations (see example in Figure 2). As soon as the antigen concentration was decreased, the direct signal was no longer detectable, whereas the enhanced signal through the vesicle binding was still detectable for significantly lower concentrations. For the dissipation curve, being sensitive to the viscoelastic behaviour of the whole vesicles, including the entrapped buffer, the detection limit – after 30 min exposure to the antigen and few minutes of vesicle adsorption – was around 5 ng/ml or 30 pM (see Figure 3B, inset). In the concentration range of interest for potential applications, the signal increased linearly with increasing antigen concentrations. The curves only started to level off for higher concentrations, i.e. *μ*g/ml, which is above diagnostically relevant concentrations of e.g. cancer markers in blood.

**Figure 4.**
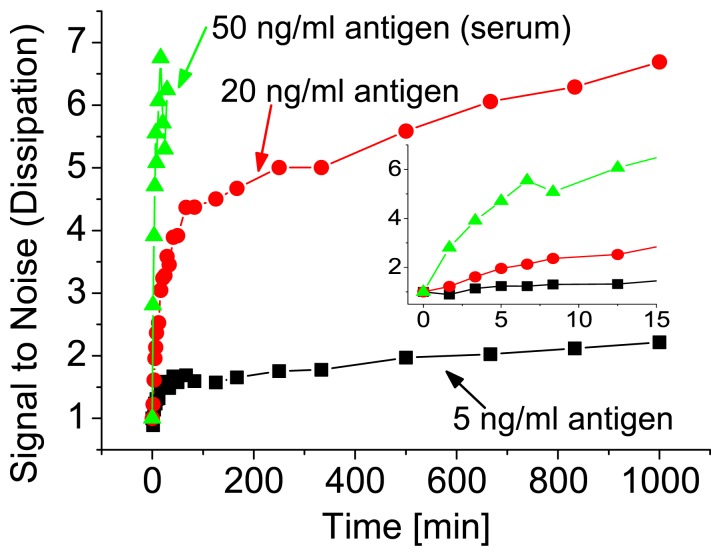
The signal-to-noise ratio of the vesicle adsorption is shown as a function of the incubation time. The inset shows a zoom in of the first 15 minutes. Note that polymeric vesicles were used for the detection from serum because of their enhanced stability.

For potential applications it is essential to obtain the result within a short time, usually within minutes. To demonstrate the assay time of our sensor, the adsorption of the vesicles as a function of incu-bation time is shown in Figure 4. The adsorption is plotted as a signal-to-noise ratio. Signal-to-noise corresponds to a dissipation change upon adsorption of the vesicles in a system with a certain antigen concentration divided by the background signal, obtained from unspecific vesicle adsorption. Thus, for no antigens it remains 1 throughout the measurement. The main increase in the signal-to-noise ratio, and therefore the response of the sensor, occurred during the first hour. Later on, the increase was only marginal. As it can be seen from the inset in Figure 4, already after few minutes a quantitative result was obtained also for the low concentration regime. For higher concentrations the curves still had the same shape, but the signal-to-noise ratio was accordingly higher. The overall sensitivity was also similar when the antigen was detected from serum (see Figure 4 (serum)). Here a concentration of 50 ng/ml antigen was spiked into 10% serum and polymeric vesicles were used because of their better stability, i.e. lipid vesicles are easily degraded by the lipase molecules present in serum.

**Figure 5.**
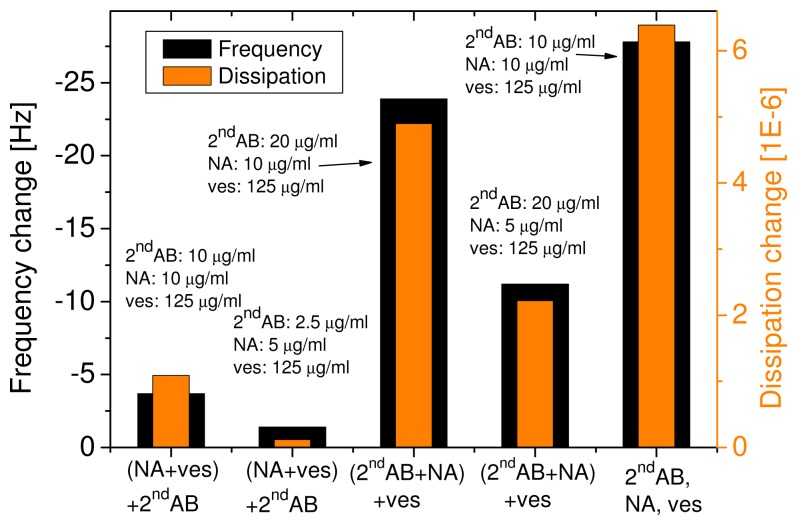
Frequency and dissipation change upon injection of NA, 2*^nd^* AB and vesicles as a function of mixing order and concentrations. For the first two measurements (from left to right) prior to injection neutravidin (NA) was first mixed with the vesicles and 10 minutes later the secondary antibody (2*^nd^* AB) was added. In the next two experiments, prior to injection NA and the 2*^nd^* AB were mixed before the addition of the vesicles. The right bar shows the control where the injection was performed in three individual steps.

To minimize the analysis time we went one step further and premixed secondary antibodies, neutra-vidin and the vesicles. The concentrations and the order in which the three reagents were mixed together were varied as shown in the example in Figure 5. The first four double-bars correspond to different variations of concentrations and mixing order, whereas the double-bar on the right shows the sum of the changes in frequency and dissipation when all three reagents were injected separately. When first NA and the vesicles were mixed for 10 min, before adding the secondary antibodies for another 10 min, the adsorption was less than 20 % compared to the single-step adsorption (see first two bars in Figure 5). The next two double-bars represent experiments where first the NA and the secondary antibody were mixed for 10 min, before the vesicles were added for another 10 min prior to injection. This mixing order turned out to be significantly better than the previously mentioned one. Depending on the ratio of NA and vesicles the achieved adsorption ranged between 40 % and 85 % of the multi-step injection.

Control experiments showed that the unspecific adsorption of premixed NA and vesicles directly on the primary antibody and BSA (without secondary antibody) was even smaller than in the normal system including the secondary antibody.

To further increase the sensitivity, vesicles with a diameter of 400 instead of 100 nm were used. For the step-by-step sensor with the larger vesicles the dissipation was increased up to 20-30 %. When the vesicles were premixed with NA or NA and secondary antibodies, no significant influence of the size was observed (results not shown).

## 3. Discussion

In this section we first describe how we were able to measure low antigen concentrations that cannot be directly detected in the QCM-D. Besides of explaining our vesicle amplification scheme we also compare our results to similar, however less sensitive, approaches that have been published during the last few years. Finally, we discuss the reduction of the assay time.

The setup of our biosensor made it possible to perform highly sensitive and reproducible measurements. Because of the comparably low mass of the antigen molecules, the signal was significantly increased by specifically binding secondary antibodies linked to vesicles. This amplification scheme enabled us to also detect antigen concentrations that did not give a sufficient signal in the QCM-D before the signal amplification through the vesicles.

At concentrations in the range of potential applications (i.e. ng/ml) the response showed a linear increase with increasing antigen concentrations before levelling off. This, together with the fact that we could reach saturation with the vesicle adsorption, suggests that for low concentrations every single antigen was detected by a vesicle, i.e. the antibodies were separated far enough so that the vesicles had sufficient space to bind to each of them. The reason why the S/N ratio curves increase fast in the beginning before levelling off could be that after a while most of the specific binding points are occupied and the fraction of unspecific binding increased for the adsorption after more than an hour. This allows also for the quantitative determination of the antigen concentration. With the currently used antibody-antigen system, the unspecific adsorption of the secondary antibody was the limiting factor. However, this is only a model system and could be further improved e.g. by additional blocking steps or by using another antibody-antigen system. The direct non-specific adsorption of vesicles or NA was negligible compared to their secondary binding through non-specifically adsorbed secondary antibodies. If we were able to lower the unspecific binding below the current limit of the detection method (e.g. by using an antibody-antigen system with a higher specificity than the current model system or by introducing blocking steps with IgG), we expect reduction of the nonspecific adsorption to further lower the detection limit.

The detection limit of the biosensor was determined by the signal to background ratio of the dissipa-tion change upon adsorption of the vesicles. With our sensor we have achieved a detection limit as low as 5 ng/ml or 30 pM. To our knowledge, so far the lowest detection limit reached by QCM-D has been published by Larsson *et al.* [13] who detected cholera toxin down to a limit of 750 pM. Thus, our new system is 25 times more sensitive than the one reported by Larsson *et al.* Furthermore, it is 160 times more sensitive than a DNA recognition sensor by Patolsky *et al.* [17] and even 300 times more sensitive than results presented by Yun *et al.* [18] some years ago.

Since time is always an issue with such sensors, we further developed our approach towards a one-pot assay. Combining the optimal concentrations and order of mixing, almost the same sensitivity was reached as by the step-by-step assay. Through this change of the protocol we were able to significantly lower the assay time. Mixing the NA and the vesicles prior to the addition of the secondary antibody resulted in a low signal. The reason could be that some of the NA molecules did not bind to a vesicle and therefore later on occupied binding sites on the secondary antibodies. On the other hand, if only few NA molecules were used, this would enhance the chance of vesicles being coupled together by NA. In other words, the vesicles would bind to a NA already coupled to another vesicle instead of binding to a free NA. Most likely, in the experiments a mixture of the two phenomena occurs. The procedure was improved by first mixing the NA and the secondary antibody before adding the vesicles. The ideal concentration mixture was found to be NA:2*^nd^* AB = 1:1 (in number of molecules). It is possible that some vesicles were still connected to each other, but this did not interfere with the measurement. As long as the mixing is always done in the same way (mixing order, concentration, mixing time), the results will be reproducible. Furthermore, this effect would even increase the sensitivity by increasing the attached mass per detected antigen.

Overall, the presented two-step assay could be performed within 30 minutes; most of them were needed for the antigen incubation. This means that, although higher vesicle concentrations could further decrease the time that is necessary for the amplification step, the limiting factor is already the adsorption of the low abundant antigen.

## 4. Conclusion & Outlook

In this paper we have described a highly sensitive, *in situ* biosensor. Unlike most existing sandwich assays we used vesicles as a mass and dissipation amplification tool instead of an enzyme-labelled secondary antibody. This allowed for detecting antigen concentrations as low as 5 ng/ml or 30 pM, which is sensitive enough to detect e.g. PSA antigens indicating prostate cancer. Compared to other sensors using the QCM-D technology, we significantly decreased the detection limit by a factor of 25 and more. Furthermore, we presented a way to decrease the assay time to about 30 miutes. With the achieved simplicity (easy to use equipment and straight-forward performance), high sensitivity as well as reduced assay time, this type of biosensor has a potential value for applications in clinical diagnostics.

## 5. Experimental Section

### 5.1. Materials

All measurements were carried out in a 10 mM 4-(2-hydroxyethyl)piperazine-1-ethane-sulfonic acid (MicroSelect, Fluka Chemie GmbH, Switzerland) solution containing 100 mM KCl. The pH of the buffer solution was adjusted to pH = 7.4. The buffer was prepared with ultrapure water (Milli-Q gradient A 10 system, Millipore Corporation, USA) and filtered (0.2 *μ*m) prior to use.

As a primary antibody, an Fc specific goat anti-mouse IgG was used. The antigen was purified mouse IgG. The secondary antibody was an Fab specific goat anti-mouse IgG conjugated to biotin. The an-tibodies were used at a concentration of 20 *μ*g/ml. They were all purchased from Sigma, Switzer-land. Serum from bovine albumin (BSA) (Sigma, Switzerland) was used to inhibit unspecific adsorption onto the surface. It was applied at a concentration of 10 mg/ml. Serum (Precinorm U) was purchased from Roche. It was 10 times diluted. NeutrAvidin (NA) (Molecular Probes, Netherlands) was used to couple biotinylated reagents together. It was used at a concentration of 10 or 20 *μ*g/ml. The vesi-cles were prepared with 1-Palmitoyl-2-Oleoyl-sn-Glycero-3-Phosphocholine (POPC) and 1,2-Dioleoyl-sn-Glycero-3-Phosphoethanolamine-N-(Biotinyl) (DOPE/biotin) lipids (Avanti Polar Lipids Inc., USA). Biotin-functionalized vesicles of poly(2-methyloxazoline)-b-poly(dimethylsiloxane)-b-poly(2-methyloxazoline) triblock copolymer [19, 20] were provided by BioCure Inc. (Norcross, GA, USA). They had a size of 200 nm and were used at a concentration of 0.1 mg/ml.

### 5.2. Methods

**The quartz crystal microbalance with dissipation monitoring (QCM-D)** measures changes in the frequency f and the dissipation factor D of an oscillating quartz crystal upon adsorption of a viscoelastic layer (Q-Sense AB, Sweden). As opposed to optical techniques, the measured mass includes hydrody-namically coupled water, water associated with the hydration layer of e.g. proteins and/or water trapped in cavities in the film [21, 22]. Therefore, the buffer volume inside the vesicles also contributes to the signal. In the results section the curves are always normalized to the 3rd overtone. QCM-D measure-ments were performed with gold coated sensor crystals (QSX 301, Q-Sense AB, Sweden). They have a 50 nm thick gold coating and a resonance frequency of 5 MHz. For more detailed information about the QCM-D technique the reader is referred to Marx *et al.* [21].

**To prepare the vesicles**, lipids, dissolved in chloroform and stored at -20°C, were used. A 99 % POPC and a 1 % DOPE/biotin solution were mixed in a round bottom flask. The chloroform was evap-orated from the lipids using nitrogen gas. Subsequently, buffer was added and the lipid layers were removed from the flask by vortexing to form big, multilamellar vesicles. When nothing else is indicated, the vesicles were 31 times extruded through polycarbonate membranes with pore sizes of 100 nm.

**The biosensor assay** is according to the scheme in Figure 1. First, a solution containing primary antibodies was injected into the flowcell. In order to inhibit unspecific adsorption, BSA was injected in a next step. Then, the antigen to be detected followed in a low concentration. Finally, the secondary antibodies, bound to vesicles via neutravidin, were injected to enhance the signal of the detected antigens. The last three steps were either performed individually or the three assay solutions were premixed prior to injection. Control experiments were run with serum. In this case, after the BSA adsorption serum was injected and the antigen was spiked directly into serum as well. Between the individual steps the system was rinsed with buffer. The vesicles adsorbed for 1 hour. For all the other steps we waited until the adsorption curve reached an equilibrium. A diagnostically relevant assay time of about 30 min was used for the adsorption of low antigen concentrations that could not be seen in the QCM-D curve.
